# The transcriptional and splicing landscape of intestinal organoids undergoing nutrient starvation or endoplasmic reticulum stress

**DOI:** 10.1186/s12864-016-2999-1

**Published:** 2016-08-26

**Authors:** Jessica Tsalikis, Qun Pan, Ivan Tattoli, Charles Maisonneuve, Benjamin J. Blencowe, Dana J. Philpott, Stephen E. Girardin

**Affiliations:** 1 Department of Laboratory Medicine and Pathobiology, Toronto, Canada; 2 Department of Molecular Genetics, Donnelly Centre, Toronto, Canada; 3 Department of Immunology, University of Toronto, Toronto, Canada

## Abstract

**Background:**

The intestinal epithelium plays a critical role in nutrient absorption and innate immune defense. Recent studies showed that metabolic stress pathways, in particular the integrated stress response (ISR), control intestinal epithelial cell fate and function. Here, we used RNA-seq to analyze the global transcript level and alternative splicing responses of primary murine enteroids undergoing two distinct ISR-triggering stresses, endoplasmic reticulum (ER) stress and nutrient starvation.

**Results:**

Our results reveal the core transcript level response to ISR-associated stress in murine enteroids, which includes induction of stress transcription factors, as well as genes associated with chemotaxis and inflammation. We also identified the transcript expression signatures that are unique to each ISR stress. Among these, we observed that ER stress and nutrient starvation had opposite effects on intestinal stem cell (ISC) transcriptional reprogramming. In agreement, ER stress decreased EdU incorporation, a marker of cell proliferation, in primary murine enteroids, while nutrient starvation had an opposite effect. We also analyzed the impact of these cellular stresses on mRNA splicing regulation. Splicing events commonly regulated by both stresses affected genes regulating splicing and were associated with nonsense-mediated decay (NMD), suggesting that splicing is modulated by an auto-regulatory feedback loop during stress. In addition, we also identified a number of genes displaying stress-specific splicing regulation. We suggest that functional gene expression diversity may arise during stress by the coordination of alternative splicing and alternative translation, and that this diversity might contribute to the cellular response to stress.

**Conclusions:**

Together, these results provide novel understanding of the importance of metabolic stress pathways in the intestinal epithelium. Specifically, the importance of cellular stresses in the regulation of immune and defense function, metabolism, proliferation and ISC activity in the intestinal epithelium is highlighted. Furthermore, this work highlights an under-appreciated role played by alternative splicing in shaping the response to stress and reveals a potential mechanism for gene regulation involving coupling of AS and alternative translation start sites.

**Electronic supplementary material:**

The online version of this article (doi:10.1186/s12864-016-2999-1) contains supplementary material, which is available to authorized users.

## Background

The cellular stress response represents a universal mechanism by which cells can sense and adapt to a variety of environmental changes. Several cellular stresses use related kinases to trigger the phosphorylation of a key residue (Ser51) in eIF2α, a translation initiation factor, resulting in global translation arrest. This cellular process is conserved from yeast to humans and is named the Integrated Stress Response (ISR). In humans, four related kinases induce eIF2α phosphorylation. General controlled nonderepressible 2 (GCN2) senses amino acid (AA) starvation, heme-regulated inhibitor (HRI) responds to oxidative stress, protein kinase R (PKR) detects viral RNA, and PKR-like endoplasmic reticulum kinase (PERK) is activated in response to endoplasmic reticulum (ER) stress [1].

A number of studies associated the pathology of severe inflammatory bowel diseases (IBD) such as Crohn’s disease and ulcerative colitis with an inability to properly adapt to the ‘stressful’ intestinal environment, resulting in unresolved mucosal inflammation. For example, studies in mice displaying a nonphosphorylatable Ser51Ala mutant eIF2α in intestinal epithelial cells (IECs) revealed that eIF2α phosphorylation is essential for proper Paneth cell function, and mutant mice displayed increased susceptibility to oral *Salmonella* infection and dextran sulfate sodium (DSS)-induced colitis [2]. Furthermore, Hamilton et al. report a marked improvement in recovery upon DSS treatment in mice lacking the transcription factor cEBP homologous protein (CHOP) – a key mediator in the ER stress-mediated apoptosis pathway induced by eIF2α phosphorylation, whereas deletion of the eIF2α kinase GCN2, which modulates T cell responses during amino acid starvation, worsened disease progression [3].

Many IBD patients present with elevated levels of ER stress markers and significant impairments within components of the unfolded protein response (UPR), which facilitates protein folding during ER stress through upregulation of molecular chaperones. In fact, variants of the UPR transcription factor gene *XBP1* have been identified as risk factors for the development of Crohn’s disease and ulcerative colitis. In addition to triggering increased autophagy and activation of ER stress markers PERK/eIF2α and ATF4, IEC-specific deletion of *Xbp1* in mice results in spontaneous enteritis, as well as Paneth cell dysfunction and hyper-inflammation in response to bacterial products such as flagellin [4].

One model for studying the link between cellular stress and gut homeostasis is premised on the remarkable capacity for self-renewal possessed by intestinal stem cells (ISCs). Clevers et al. demonstrated that a single adult ISC positive for the marker *Lgr5* could generate a self-organizing stratified epithelium consisting of all the major cell lineages of the gut when cultured on a glycoprotein substratum (Matrigel^TM^) in condition media supplemented with appropriate growth factors [5]. Specifically, ISCs differentiate into 3D organ-resembling structures or ‘organoids’, which are comprised of villi and crypts, with crypt-residing Lgr5^+^ stem cells, highly proliferative undifferentiated transit-amplifying (TA) cells and differentiated cells (Paneth, enterocytes, goblet, enteroendocrine and tuft cells). Recently, the link between ER stress and ISC self-renewal was highlighted by the finding that differentiated villus cells display high levels of ER stress, measured by expression of various UPR components, and that this induction of ER stress causes a rapid loss of the stem cell signature in a PERK/eIF2α-dependent manner [6].

In addition to changes in the landscape of transcript expression upon stress, there is increasing evidence that alternative splicing (AS) enhances the ability of cells to cope with various stresses. In plants, genome-wide RNA sequencing (RNA-seq) based approaches have highlighted a role for AS as a key mode of generating genome plasticity during abiotic stresses such as osmotic shock, thermal shock and drought (Reviewed in [7]). Interestingly, many stress-regulated AS events in both plants and mammals are associated with genes encoding splicing factors and various RNA processing proteins [8–10]. Furthermore, several studies have shed light on the effect of cellular localization of core spliceosomal factors such as hnRNP A1 on pre-mRNA splicing during oxidative stress and heat shock [11, 12]. Despite expanding knowledge in the field, a comprehensive global analysis of how the AS landscape is modulated during stress in mammalian systems is lacking.

Here, we provide the first genome-wide characterization of the impact of ER stress and nutrient starvation on both the steady-state transcript levels and AS landscape in primary murine organoid cultures. Our results highlight the core response to ISR-associated stress, which includes the prominent induction of stress transcription factors and inflammatory genes. Furthermore, we observed that ER stress and nutrient starvation surprisingly had opposite effects on ISC reprogramming and proliferation. Our analysis of AS highlighted the common role of ER stress and nutrient starvation in regulating splicing of detained introns in splicing genes, likely revealing a homeostatic level of feedback control of the splicing machinery. In addition, many of the reported AS events were stress-specific. Our analysis revealed that stress-specific pools of genes might be regulated by the coordination of alternative splicing and alternative translation to generate functional diversity, and this mechanism might represent a previously unrecognized mechanism of cellular response to stress. Because these analyses were performed in primary intestinal organoids and not in a cancer cell line, our results provide a faithful analysis of the genome-wide effect of the ISR in a near-physiological context of growing intestinal crypts. Together, this study provides insights into the importance of cellular stresses in the regulation of immune and defense function, metabolism, proliferation and ISC activity in the intestinal epithelium.

## Methods

### Crypt isolation and organoid culture

To generate organoid cultures, crypts from the small intestines of wildtype mice (12 total) were extracted as previously described [5]. Briefly, the villi of the small intestine were removed by scraping, followed by washing with cold PBS. The remaining tissue were then homogenized and incubated in 2 mM EDTA in PBS for 30 min at 4 °C, followed by vigorous washing in PBS several times to obtain crypt-enriched supernatant fractions. The supernatant fractions were then passed through a cell strainer, pelleted at 300 g for 5 min at 4 °C and resuspended in 50 ul Matrigel (Corning). The crypt-containing organoids were cultured by plating onto the center of a 24-well plate and grown in 500 ul crypt culture medium supplemented with growth factors (R-spondin 1, Noggin, EGF). Organoids were allowed to grow 7 days, followed by passaging onto 6-well plates for stimulation (roughly 10-15 isolated organoids). To prepare samples for RNA-seq, organoids were left either unstimulated or treated with 5 uM thapsigargin (Sigma-Aldrich) or Krebs Ringer Bicarbonate (KRB) buffer for nutrient starvation conditions (118.5 mM NaCl, 4.74 mM KCl, 1.18 mM KH_2_PO_4_, 23.4 mM NaHCO_3_, 5 mM glucose, 2.5 mM CaCl_2_, and 1.18 mM MgSO_4_, pH 7.6) for 4 h. Samples were then pelleted by centrifuging at 1000 RPM for 10 min and homogenized using the QIAshredder according to the manufacturer’s protocol.

### RNA-seq library preparation and sequencing

Total RNA was then extracted from the organoid samples using the GeneJET™ RNA Purification Kit (Thermo Scientific) according to the manufacturer’s protocol. Eluted RNA was treated with DNAse I (Fermentas) at 37 °C to remove genomic DNA. Triplicate samples containing 4 ug of RNA extracted from control vs. thapsigargin treated (5 mM for 4 h) and control vs. nutrient starved (4 h) primary murine enteroids were submitted for RNA-seq at the Donnelly Sequencing Center at the University of Toronto. Illumina TruSeq V2 mRNA libraries were generated, followed by ~100 million 100-bp paired end reads via Illumina HigSeq2500. Three replicates for each condition were sequenced. Alternative splicing analysis was performed essentially as previously described [13, 14]. In brief, RNA-Seq reads were first aligned to canonical transcript sequences, and reads mapping to more than one location were removed. The remaining reads were mapped to a database of mouse splice junctions, allowing up to two mismatches/indels (insertions or deletions). The percent inclusion, or “percent spliced-in” (PSI) value, and percent intron retained (PIR) value for each internal exon and intron, respectively, was calculated as previously described [14, 15]. 

### Gene expression analysis

GO analysis was conducted to assess gene enrichment upon thapsigargin treatment or nutrient starvation, analyzed from the Sanger database for GO terms. Genome wide analysis resulted in a manually curated list of 8 groups (Group 1 – Cell Signalling, Group 2 – Channels/Transporters, Group 3 – Cytoskeleton, Group 4 – Gene Expression, Group 5 – Metabolism, Group 6 – Receptors/PM/ECM, Group 7 – Secreted, Group 8 – Uncertain), which were used for subsequent expression analyses. Scatterplot expression graphs were generated using GraphdPad Prism software, red lines designate genes expressed more or less than 2.5-fold.

### Proliferation assay

Organoid cell proliferation was measured by flow cytometry using the Click-iT EdU AlexaFluor647 Flow Cytometry Assay Kit (Life Technologies).

### Semi-quantitative RT-PCR validation of AS events

Following RNA extraction, genomic DNA contaminants were removed via treatment with DNAse 1 for 45 min at 37 °C. Purified RNA was then reverse transcribed to cDNA using SuperScript III MMLV reverse transcriptase (Invitrogen) with random hexamers and oligo-dT primers. The cDNA was diluted appropriately and used as template for semi-quantitative PCR using Phusion polymerase according to manufacturer’s protocol using primers specific for AS events (see Table 1). PCR products were then run on an agarose (3 %) gel and visualized using Gel Doc 2000 Gel Imaging System (BioRad).

**Table 1:** Murine RT-PCR primers used for AS event validation in this study

Gene Name	Primer Sequences (5’- 3)’
*Hnrnpd*	FW: GAAAGTATCCAGGCGAGGTG
	RV: GCTATTAGCAGGTGGCAGGA
*Hnrpdl*	FW: CAGACTACAGCGGTCAGCAG
	RV: TGGACCAATACCCCCTACAA
*Srsf7*	FW: CGCCTTGATTCAGAATGTCA
	RV: TGATCTTGACCTCCGTCCTC
*Ogt (partial intron 4 retention)*	FW: ACTGTGTTCGCAGTGACCTG
	RV: CAAATCTCCCCTTGTGCATT
*Ogt (full intron 4 retention)*	FW: CTTGGTAGCAGCAGGTGACA
	RV: AATGCTCACGGTCTTGCTTT
*Slc35b1*	FW: AAGGACCCAAACAGGAGACA
	RV: ATGGCACCCACATAGGAGAC
*Ufd1l*	FW: TGTTCATTTTATTTCAAAAATCGGAGC
	RV: AAGAACTCATCATAGTGCTCCTGC
*Ivnsabp1*	FW: AGCATCTGGGAGAATGGAGA
	RV: CATCATCACTGCCAAACACC
*Casp4*	FW: TGCTGAACGCAGTGACAAGC
	RV: TAAGAGCCTTTCGTGTACGGC
*Nnt*	FW: AACAGTGCAAGGAGGTGGAC
	RV: GTGCCAAGGTAAGCCACAAT
*Nt5c3*	FW: GCTGGCCCAGTACATATTCA
	RV: GGGCATCTTTTCCCATTGTA
*Frrs1*	FW: TTGCATTTCTCACGACCAG
	RV: TAGCCTCAGGAAGGGTGATG

### Statistical analysis

Results are expressed as means ± s.e.m of data obtained in independent experiments. Significant differences between mean values were evaluated using a one-sample or unpaired t-tests. * indicates *p* < 0.05, ** indicates *p* < 0.01 and *** indicates *p* < 0.001.

## Results

### RNA-seq data collection of primary intestinal epithelial organoids undergoing ER stress or nutrient starvation

**Fig. 1:**
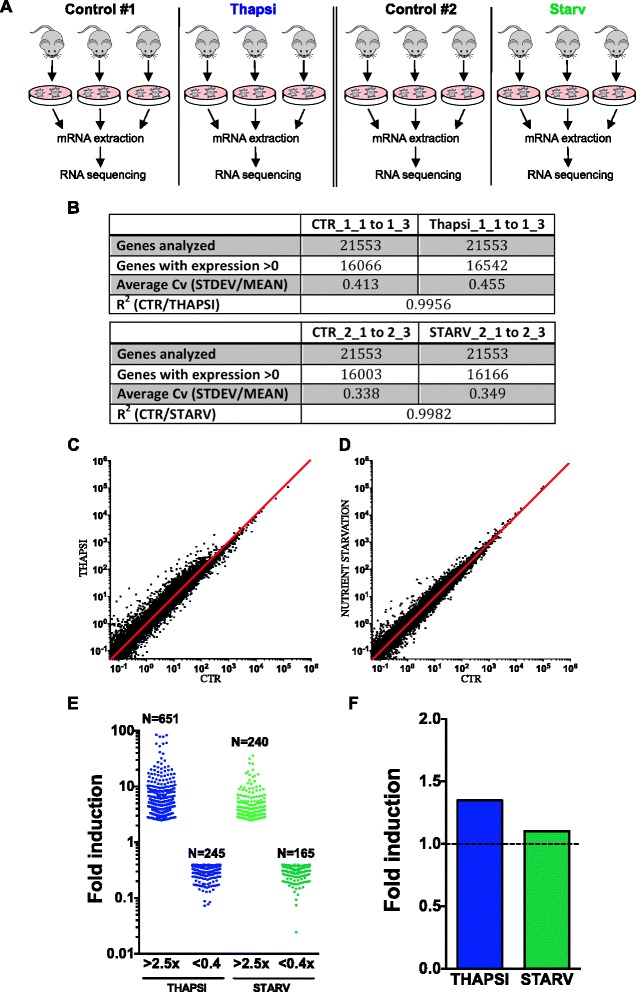
RNA-seq analysis of transcript levels in thapsigargin-stimulated and nutrient-starved murine enteroids. **a** Experimental design of samples submitted for RNA-seq. Intestinal organoid cultures were derived from three separate mice per condition. RNA was purified from organoids stimulated with either thapsigargin or nutrient-starved for 4 h and sent for RNA-seq. **b** Overview of the total number of genes analyzed (average over three replicates), including the adjusted total number of genes excluding those which had RPKM values >0. **c**, **d** Scatterplot analysis of the log10 changes in expression of the total (adjusted for RPKM values >0) number of genes in thapsigargin versus control enteroids (**c**) and nutrient starved versus control enteroids (**d**). **e** Violin scatter boxplot analysis of the genes induced and repressed more than 2.5 fold upon thapsigargin treatment and nutrient starvation. **f** Average fold change overall in the genes analyzed upon thapsigargin and nutrient starvation

In order to gain a global understanding of how IECs are regulated by stress, RNA-seq analysis was first conducted on mRNA extracted from small intestinal organoid cultures from six mice either left unstimulated or treated with thapsigargin, a molecule that induces ER stress (Fig. 1a). In a second set of experiments, intestinal organoids from six other mice were used to analyze by RNA-seq the impact of nutrient starvation on IEC gene expression programs. For both stresses, we chose to analyze an early response (4 h stimulation), to concentrate on immediate cellular responses to these stresses and limit the impact of potential regulatory feedback loops that could alter gene expression programs in these organoids in a paracrine or autocrine-dependent manner. Moreover, analyzing early responses to these stresses limited the potential impact of cell death, which was undetectable under our experimental conditions at this early time of stimulation (data not shown).

Gene expression analysis was performed using the RPKM (Read Per Kilobase of transcript per Million) normalization method. A total of 21,553 genes were analyzed per condition, of which approx. 16,000 had detectable (>0) expression in organoids in the experimental conditions analyzed (Fig. 1b and Additional file 1: Table S1). In agreement with the role of the intestinal epithelium in nutrient absorption and host defense, the genes most expressed in our organoid cultures were associated with metabolism (including *Aldoa*, *Aldob*, *Mt1*, *Mt2*, *Apoa1*, *Ldha*) and innate immunity (*Gm15284*, *Defa24*, *Lyz1*) (Additional file 2: Table S2). Global analysis showed that, for each experimental condition, the triplicate cultures of organoids displayed overall very robust consistency in gene expression, with average correlation coefficients (standard deviation between triplicates divided by the mean for each gene analyzed) ranging between 0.338 to 0.455 (Fig. 1b). The coefficient of determination (R^2^) for both datasets (CTR_1 versus Thapsi_1 and CTR_2 versus Starv_2) was close to 1 (0.9956 and 0.9982, respectively), thus indicating that little experimental noise was generated in our assays and that most genes were not extensively altered at the transcript level by the stimulations (Fig. 1b-d). Nevertheless, 651 and 245 genes were found to be upregulated (>2.5x) and down-regulated (<0.4x) in thapsigargin-stimulated organoids, respectively, while 240 and 165 genes were found to be upregulated (>2.5x) and down-regulated (<0.4x) in nutrient starved organoids, respectively (Fig. 1e and Additional file 2: Table S2). On average, genes expressed in intestinal organoids were upregulated 1.35x and 1.10x by thapsigargin and nutrient starvation, respectively (Fig. 1f).

### Transcript level analysis of murine intestinal epithelial organoids undergoing ER stress

**Fig. 2:**
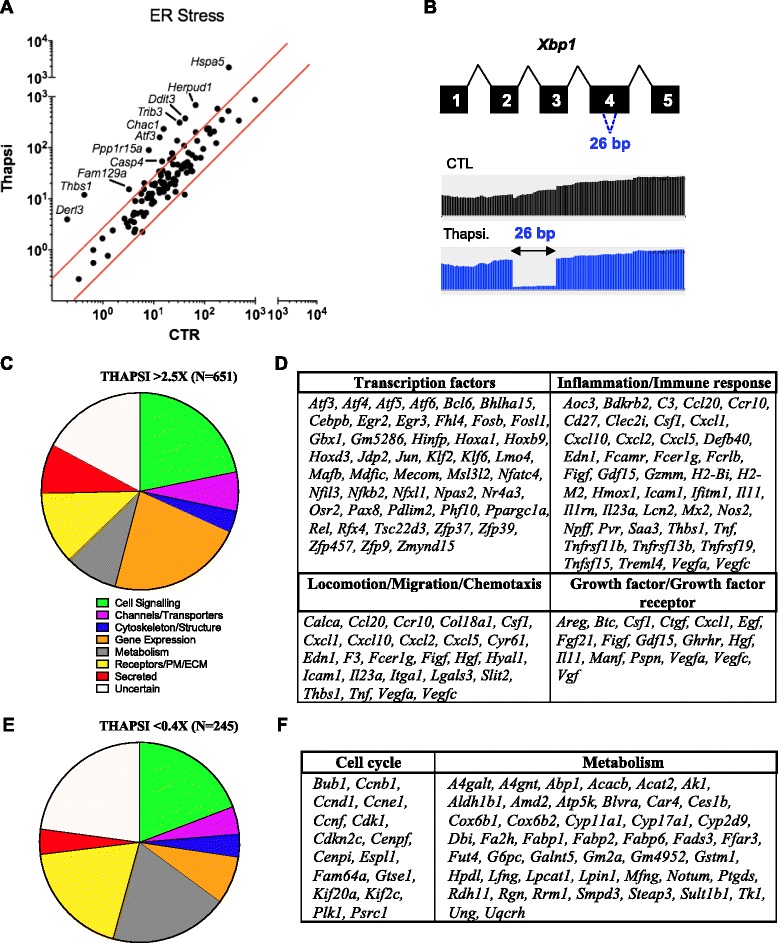
Induction of an ER stress transcriptional program in thapsigargin-stimulated enteroids. **a** Scatterplot of the expression of ER stress response classified genes. Genes above the top red line represent a 2.5-fold increase in expression, while genes below the bottom red line represent genes down-regulated 2.5-fold. **b** Alternative splicing of transcription factor gene *Xbp1* upon thapsigargin treatment, visualized via integrated genome browser (IGV). **c** Venn diagram analysis of 651 genes upregulated more than 2.5 fold upon 4 h of thapsigargin treatment, categorized by gene function. **d** Gene list manually curated based on gene function from the group of 651 upregulated genes. **e** Venn diagram analysis of 245 genes down-regulated more than 2.5 fold upon 4 h of thapsigargin treatment, categorized by gene function. **f** Gene list manually curated based on gene function from the group of 245 downregulated genes

We first analyzed the transcriptional response to thapsigargin in primary intestinal organoids. As expected, the transcriptional landscape shaped by thapsigargin treatment displayed a very strong (*p* = 1.6 × 10^-12^) ER stress-associated signature, and Gene Ontology (GO) analysis revealed that GO #0034976 (“Response to endoplasmic reticulum stress”) was the most significantly associated GO group among genes upregulated >2.5 fold by thapsigargin. ER stress related genes such as *Atf3*, *Chac1*, *Thbs1*, *Ddit3*, *Derl3*, *Herpud1* and *Hspa5* were upregulated more than 2.5 fold (Fig. 2a). Furthermore, the ER stress and UPR-associated transcription factor *Xbp1*, which is alternatively spliced upon ER stress, clearly underwent splicing to generate the exon 4 Δ26bp ER stress-specific isoform (Fig. 2b), and its expression was upregulated 2.5 fold. Thus, thapsigargin stimulation resulted in a strong induction of ER stress-associated responses in intestinal organoids.

Analysis of the 651 genes induced by thapsigargin over 2.5 fold revealed that, in addition to the core ER stress reprogramming at the transcript level, genes associated with cellular signaling (Group 1), gene expression regulation (Group 4), metabolism (Group 5) and the cytoskeleton (Group 3) were upregulated, as well as genes encoding channels and transporters (Group 2), cell surface molecules, receptors and extracellular matrix proteins (Group 6) and secreted factors (Group 7) (Fig. 2c). Therefore, ER stress induction by thapsigargin triggers a global transcriptional adaptation in intestinal organoids that affects multiple cellular processes and pathways.

Interestingly, a number of inflammation-associated genes were upregulated and were found mainly in Groups 6-7. These include the chemokines *Cxcl10*, *Cxcl5*, *Cxcl1* and *Ccl20*, the cytokines *Il23a* and *Tnf*, the acute-phase response factor *Saa3* and other genes encoding secreted mediators associated with inflammation, such as *Fgf21* and *Lcn2*, as well as genes associated with the cellular adaptation to an inflammatory milieu (*Hmox1*) or innate immunity (*Ifitm1*, *Nos2*, *Mx2*, *Defb40*) (Fig. 2d). Thapsigargin stimulation also upregulated the expression of *Fut1*, a gene responsible for the fucosylation of extracellular matrix glycoproteins, an event that has recently been shown to play a major protective role in the response to bacterial pathogens in the small intestine [15]. Thus, in the intestinal epithelium, the cellular response to the ER stress inducer thapsigargin bears striking similarities to the inflammatory response against microbial infection.

Thapsigargin stimulation also upregulated genes associated with chemotaxis, migration and locomotion as well as genes encoding growth factors or growth factors receptors (Fig. 2d), which were found in Groups 6-7. Such growth factors included *Hgf*, *Egf*, *Ctgf*, *Gdf15*, *Manf*, *Vegfa*, *Vegfc* and *Vgf*. This might be of physiological importance given the known contribution of ER stress in the modulation of cancer microenvironment and tumor growth [16].

Global transcriptional reprogramming commonly requires upregulation of a core group of transcription factors (TFs) as part of the first induction wave of so-called “immediate early genes”. In agreement, genes associated with Gene Expression Regulation (Group 4) represented the largest group of genes up-regulated by thapsigargin (*N* = 145) and among those genes, a remarkable number (*N* = 46) of TFs were identified (Fig. 2d). Moreover, TFs were also found among the genes that are down-regulated by thapsigargin, including *Mycn* and *Myb* as well as the Notch target *Hes5*. Interestingly, Notch-dependent genes and in particular *Hes* family members play key roles in intestinal cell fate decision and proliferation, suggesting a potential role of ER stress signaling in IEC lineage commitment through *Hes5* down-regulation (see also below). Together, these immediate-early TFs likely play a critical role in shaping the global cellular transcriptional adaptation to ER stress.

Thapsigargin stimulation also down-regulated the expression of a number of genes in intestinal organoids (*N* = 245 for genes down-regulated >2.5 fold = regulated <0.4 fold) (Fig. 2e). Of particular significance, we noticed that genes involved in metabolism (Group 5) were over-represented when compared to upregulated genes, suggesting that thapsigargin stimulation results in potent down-regulation of major cellular metabolic pathways (Fig. 2f). Moreover, among the genes associated with cellular signaling (Group 1), we noticed that a number encode for proteins associated with cell cycle regulation (Fig. 2f), suggesting a direct impact of thapsigargin on cellular proliferation (see also below, Fig. 4). Together, this analysis identified the core transcriptional program regulated by ER stress in primary intestinal organoids.

### Transcript level analysis of murine intestinal epithelial organoids undergoing nutrient starvation

Primary murine organoids require a complex mixture of growth factors and nutrients to proliferate and to undergo differentiation of their stem cells into the different absorptive or secretory lineages of a functional intestinal epithelium. We chose to perform a short-term nutrient starvation by replacing the normal organoid culture medium with a Krebs-Ringer bicarbonate (KRB) buffer that lacks growth factors and AAs, which should result in acute inhibition of mTOR signaling and induction of the GCN2-dependent arm of the ISR. Under these conditions, although starvation resulted in the regulation of substantially fewer genes than thapsigargin (see above Fig. 1e), we noticed that the relative proportions of upregulated genes from Groups 1 to 7 were comparable between the thapsigargin and nutrient starved organoids (Additional file 3: Figure S1A). Similar to thapsigargin-treated organoids, a number of TFs, genes associated with inflammation, chemotaxis and growth factor signaling were identified in nutrient starved organoids (Additional file 3: Figure S1B). GO #0006935 (chemotaxis) was the most significantly associated GO term for genes upregulated >2.5 fold by nutrient starvation. With regards to genes down-regulated by nutrient starvation, 41.8 % (69/165) were genes whose function was uncharacterized (Additional file 3: Figure S1C), which is much greater than the percentage of uncharacterized thapsigargin-repressed genes (22.9 %) (Additional file 3: Figure S1D), suggesting that a significant portion of the cellular response to nutrient starvation in intestinal organoids involves poorly characterized pathways and processes. Nevertheless, among the genes with an attributed function, we noticed that much fewer were associated with cell cycle regulation as compared to genes down-regulated by thapsigargin (Additional file 3: Figure S1E), suggesting that ER stress and nutrient starvation affect cell cycle regulation in a distinct manner (see also Fig. 4 below). Finally, we observed that a number of genes associated with cellular innate immunity (*Card11*, *Ifitm6*, *Mx2*, *Nlrp10*, *Nlrp1b*, *Nos3* and *Noxa1*) were downregulated by nutrient starvation, which contrasts with results obtained in thapsigargin-stimulated cells.

Together, we provide a comprehensive analysis of the modulation of the transcriptional landscape by nutrient starvation and reveal global similarity/dissimilarity patterns as compared to the regulation occurring in cells undergoing ER stress.

### Identification of a common transcriptional signature for ER stress and nutrient starvation

**Fig. 3:**
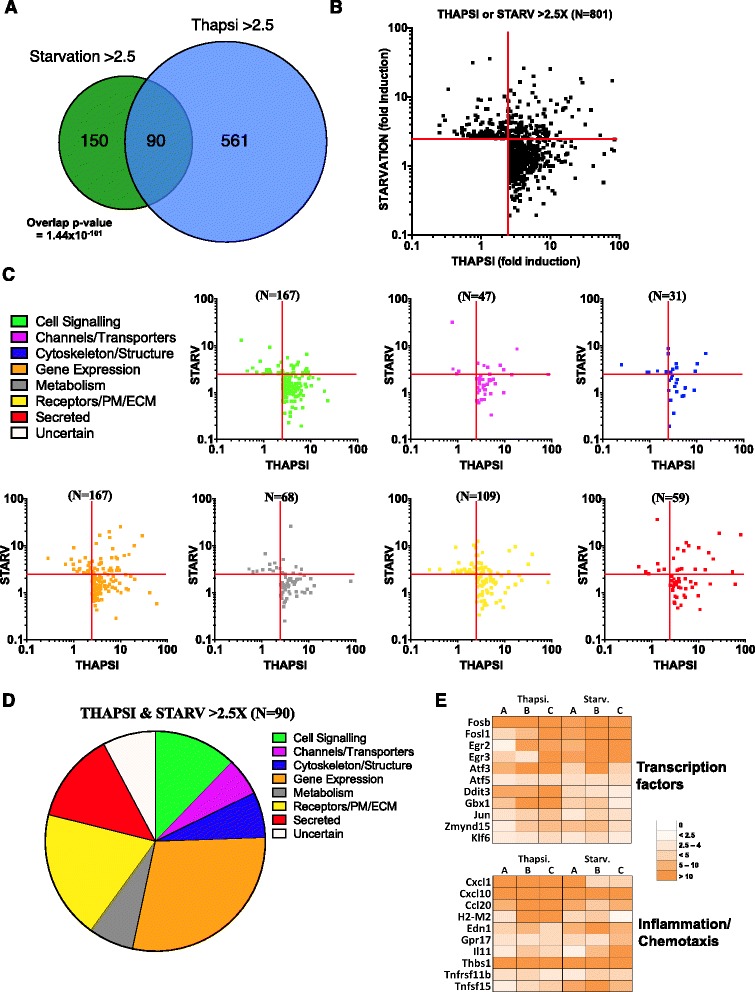
Nutrient starvation and ER stress induce a common transcriptional landscape in murine enteroids. **a** Venn diagram comparison of the genes commonly upregulated more than 2.5 fold upon nutrient starvation and thapsigargin treatment. Hypergeometric tests were used to calculate the *P* values for significance of overlaps. Scatterplot analysis of the genes transcriptionally upregulated (cutoff >2.5 fold) upon thapsigargin and nutrient starvation. Plots are displayed analyzing of the total number of genes (801) (**b**) or categorized by gene function (**c**). **d** Analysis of the gene function of the 90 genes commonly upregulated upon thapsigargin and nutrient starvation. **e** Expression profiles of genes encoding transcription factors and genes involved in inflammation/chemotaxis, manually picked from the 90 genes upregulated more than 2.5 fold upon thapsigargin/nutrient starvation. Expression profiles of three biological replicates per stimulation are shown (lanes **a**, **b** and **c**)

We aimed to take our analysis one step further and to identify the common transcriptional signature associated with these two ISR-triggering cellular stresses. Focusing first on upregulated genes, we found that a core group of 90 genes were upregulated by both stresses (Fig. 3a). Considering the pool sizes (*N* = 651 and *N* = 240 for thapsigargin stimulation and nutrient starvation, respectively), this intersection was found highly significant (*p* = 1.44×10^-101^), thus showing that a strong common transcriptional signature exists for genes upregulated by these two stresses. It must be noted that these common genes represented a smaller fraction of the thapsigargin upregulated pool (90/651 = 13.8 %) than the nutrient starvation upregulated pool (90/240 = 37.5 %), thus showing that in our experimental conditions, thapsigargin induced a wider spectrum of stress-specific genes than nutrient starvation. Consistent with this, a large majority of the genes induced by thapsigargin were not significantly modulated by nutrient starvation (Fig. 3b).

When upregulated genes were separated in functional categories, it appeared that most of the genes upregulated by both stresses were found in Groups 4 (“Gene expression”), 6 (“Cell surface molecules, receptors and extracellular matrix proteins”), 7 (“Secreted”) and 1 (“Cellular signaling”) (Fig. 3c-d). Among the 90 common genes, those that were most upregulated by both conditions included TFs (found in Group 4) and factors associated with inflammation and/or chemotaxis (in Groups 6/7) (Fig. 3c and e).

The analysis of down-regulated genes revealed that few genes (*N* = 18) were found to be down-regulated by both stresses (Additional file 4: Figure S2A-B), and these genes were either of uncertain function (7/18) or had unrelated functions (Additional file 4: Figure S2C-D).

Together, these results demonstrate the existence of a common transcriptional signature associated with two distinct ISR-inducing stresses. While a core group of 11 TFs likely promotes this transcriptional reprogramming, genes associated with inflammation and chemotaxis were unexpectedly also part of this common transcriptional signature.

### Effect of cellular stress on intestinal stem cells and proliferation

**Fig. 4:**
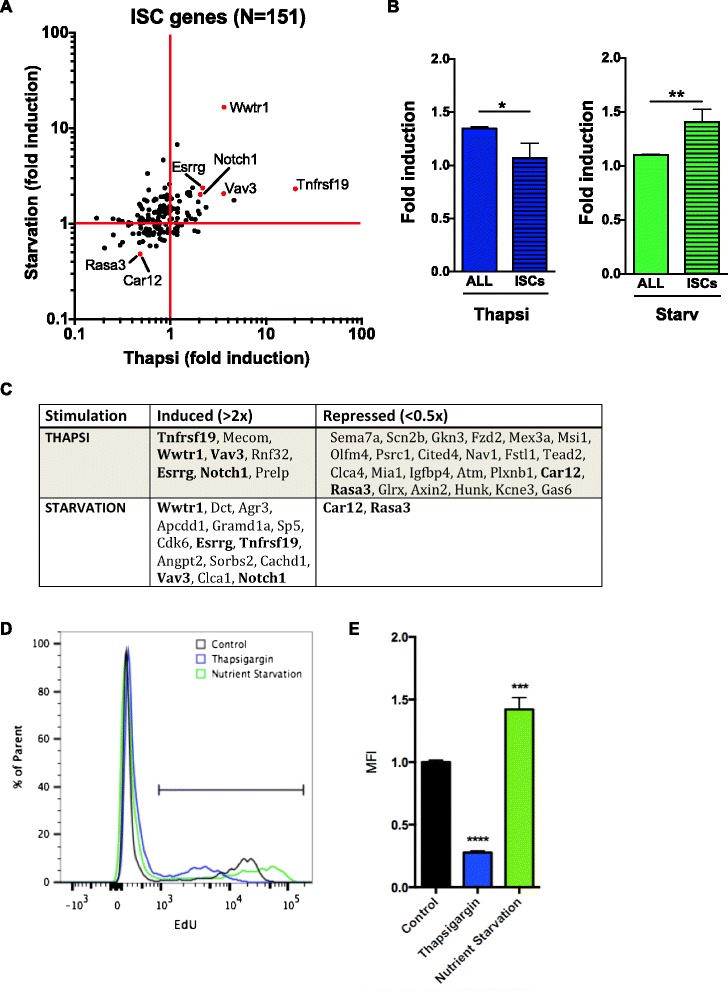
Metabolic stress affects the expression profile of intestinal stem cell genes. **a** Scatterplot analysis of the fold expression profiles of 151 intestinal stem cell (ISC) genes upon thapsigargin and nutrient starvation. **b** Overall fold induction of ISC genes compared to the total gene induction upon thapsigargin treatment and nutrient starvation. **c** List of ISC genes upregulated and downregulation more than 2 fold upon stress. **d** Cell proliferation was analyzed by flow cytometry by monitoring EdU incorporation during the last 2 h of the 4 h treatment. Representative profiles of thapsigargin-treated (*blue*), nutrient-starved (*green*) and untreated control organoids (*black*) are shown. **e** Quantification of cell proliferation is presented as mean fluorescence intensity (MFI)

In primary intestinal organoids, cell proliferation is dependent on the activity of ISCs and ISC-derived transit-amplifying cells that express the marker Lgr5 and occupy an important fraction of the intestinal crypt [17]. The above results suggested that ER stress and nutrient starvation affected the expression of cell cycle and/or proliferation genes differentially in intestinal organoids (see Fig. 2 and Additional file 3: Figure S1). Therefore, to identify the overall impact of thapsigargin versus nutrient starvation on ISCs, we analyzed how these stresses affected the transcript levels of the 151 ISC-enriched genes identified recently by sorting Lgr5^+^ intestinal epithelial cells [18]. Interestingly, the majority of ISC genes displayed reduced expression following thapsigargin treatment (fold induction <1), while the opposite result was observed in nutrient starved cells (Fig. 4a). Overall, expression of ISC genes was not upregulated (1.06 fold) by thapsigargin while this treatment upregulated gene expression 1.34 fold genome-wide (Figs. 1f and 4b). In contrast, ISC genes were significantly more upregulated (1.41 fold) than all genes (1.1 fold) by nutrient starvation (Figs. 1f and 4b). Despite these differences, we observed that a group of seven genes were similarly modulated by both stresses: *Tnfrsf19*, *Wwtr1*, *Vav3*, *Esrrg* and *Notch1* were down-regulated by thapsigargin and nutrient starvation, while *Car12* and *Rasa3* were upregulated. These seven genes might thus represent the core group of genes involved in the cellular adaptation of ISCs to stress.

Finally, we aimed to directly test the differential effect of thapsigargin and nutrient starvation on cellular proliferation in intestinal organoids by measuring EdU incorporation in organoids undergoing stress for 4 h. In agreement with the ISC gene expression data, we observed that thapsigargin stimulation resulted in potent reduction of EdU incorporation in organoids, indicative of a block in proliferation, while nutrient starvation had the opposite effect (Fig. 4d-e). Thus, while ER stress affects ISC gene expression and proliferation in a way that is expected for a cellular stress and is in line with previous results [6], short-term nutrient starvation appeared to trigger an apparently paradoxical pro-proliferative response.

### Analysis of the AS landscape in enteroids undergoing ER stress and nutrient deprivation

**Fig. 5:**
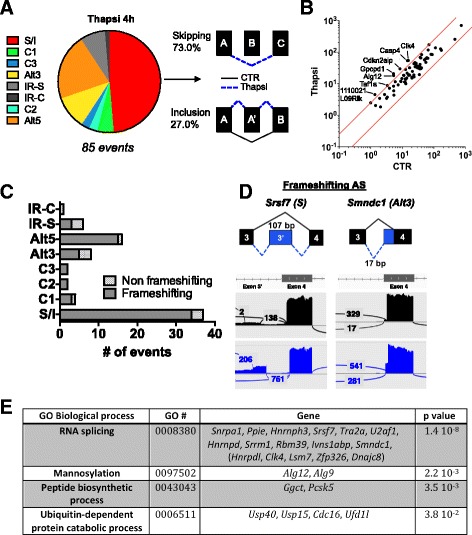
Analysis of the alternative splicing landscape in murine enteroids upon thapsigargin treatment. **a** Classification of AS events induced upon thapsigargin based on type of splicing event (skipping(S)/inclusion(I), complex 1 (C1), complex 3 (C3), alternative 3’ (Alt3), intron retention simple (IR-S), intron retention complex (IR-C), complex 2 (C2), alternative 5’ (Alt5)). Examples of both skipping and inclusion events are shown. **b** Scatterplot of the expression of the genes found to undergo AS upon thapsigargin treatment. Genes above the top red line represent a 2.5-fold increase in expression, while genes below the bottom red line represent genes down-regulated 2.5-fold. **c** Comparison of the proportion of frameshifting vs. non-frameshifting events within each category of AS type. **d** Examples of frameshifting AS events in Srsf7 (S) and Smndc1 (Alt3). Gene schematics showing the AS events in blue, as well as the Sashimi plots obtained by IGV showing the total read numbers for each junction. **e** GO analysis of the gene group enrichment among the genes that underwent AS during thapsigargin treatment

One key aim of this study was to gain insight into the role of AS on gene regulation during metabolic stress – an idea that has been understudied in mammalian systems. In order to investigate global changes in AS occurring upon nutrient starvation and ER stress, AS events were analyzed from the RNA-seq dataset using criteria that has previously been described [13]. Applying a stringent cutoff (*p* ≥ 0.90, *DBS* = 0.1, *RN* = 5, *See**Methods*) to identify high confidence AS events, we identified a group of 85 AS events that were significantly upregulated by thapsigargin treatment and 42 AS events induced by nutrient starvation (Fig. 5a, Additional file 5: Figure S3A and Additional file 6: Table S3). When classifying the AS events by type of splicing event, the largest group during ER stress corresponded to exon skipping (S/I) events, followed by an approximately 25 % proportion being alternative 5’ or 3’ donor/acceptor changes, with complex events (C3, C2, IR-C) representing the smallest percentage. During nutrient starvation however, we observed a more even distribution among S/I events and events involving full intron retention (IR-S), as IR-S and complex intron retention (IR-C) events comprised more than half of the observed AS events. To rule out that the increase in AS events could be attributed to simply an upregulation in transcription, we analyzed the expression levels of the genes found to undergo AS upon ER stress or nutrient starvation. Only a handful of genes were found to be upregulated at the transcript level (*Casp4, Clk4, Cdkn2aip, Gpcpd1, Alg12, Taf1a*, and *1110021L09Rik* for ER stress and *Glipr1* and *Maff* for nutrient starvation), while the rest of the genes underwent no significant change in expression (Fig. 5b, Additional file 5: Figure S3B).

Interestingly, closer examination of the thapsigargin-regulated AS events displaying the highest confidence score in our analysis (*p* ≥ 0.95, *DBS* = 0.15, *RN* = 10) revealed that the vast majority of those events were predicted to produce a reading frame shifting caused by exon inclusion, skipping or alternative 5’/3’ splice site usage (Fig. 5c). Upon nutrient starvation conditions however, we observed that the proportion of AS events that resulted in frame shifted reading frames (29 out of 42 events = 69 %) was roughly that which would be expected by chance (2/3 = 66.6 %) (Additional file 5: Figure S3C). The predicted stress-induced AS events that met our cutoff criteria were visualized using Integrated Genome Viewer (IGV) (Fig. 5d and Additional file 5: Figure S3D).

Gene ontology analysis of the most enriched gene family among the genes undergoing AS during both ER stress and nutrient deprivation revealed a significant enrichment for genes involved in mRNA splicing (*p* = 1.4x10^-8^), including the U snRNA maturation-associated factor *Lsm7,* the SR protein *Srsf7* and the *Smn* paralog *Smndc1* (Fig. 5e and Additional file 5: Figure S3E), providing further support for our results that identified a key role for metabolic stress stimuli in the dynamic regulation of the splicing machinery [19].

### Increased expression of PTC-containing AS isoforms of RNA processing/splicing genes upon metabolic stress

**Fig. 6:**
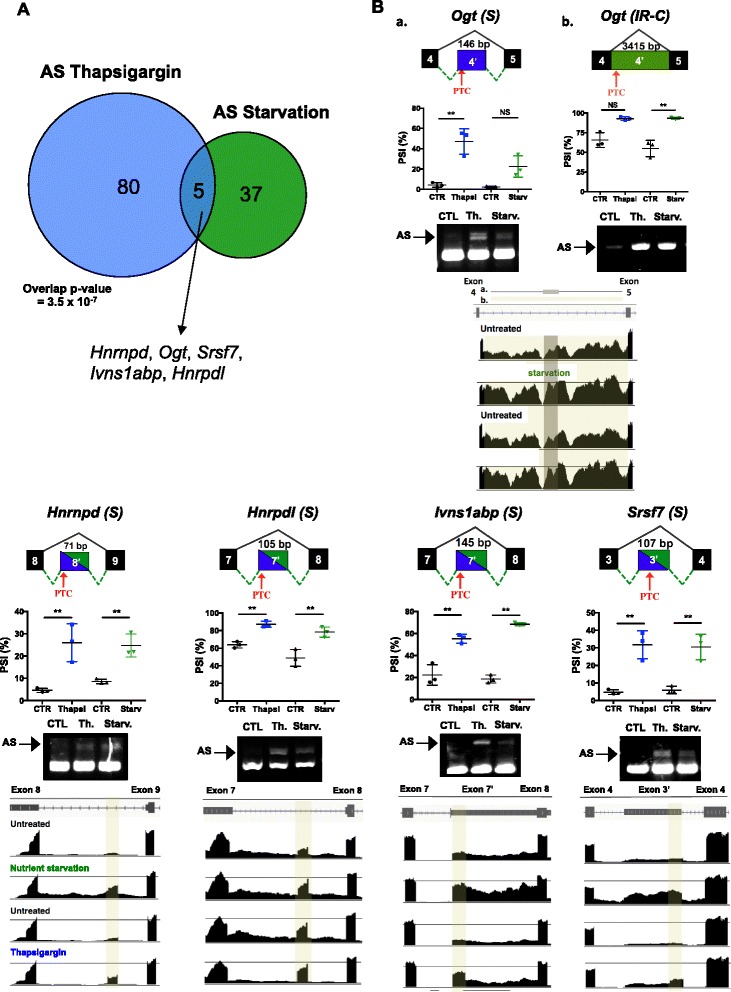
ER stress and amino acid starvation induce a common alternative splicing signature. **a** Venn diagrams demonstrating the overlap between the AS events occurring during thapsigargin treatment and nutrient starvation. The overlap p value was calculated using a hypergeometric test. **b** Validation of AS events for *Hnrnpd, Ogt, Srsf7, Ivns1abp, Hnrpdl.* Gene schematics highlighting the AS events along with approximate location of the premature termination codon (PTC), along with PSI value plots for each event. Semi-quantitative RT-PCR validations for each event are shown, with the spliced PCR product labeled by an arrow. IGV plots of RNA-seq read for each AS event and adjacent sequence reads, with AS events highlighted accordingly

As mentioned above, GO analysis of the genes undergoing both thapsigargin- and nutrient deprivation-upregulated AS revealed a significant enrichment in genes associated with mRNA splicing regulation. Using the cut-off criteria outlined earlier, we observed a significant overlap (*p* = 9.6 × 10^-8^) of the AS events in splicing factors during nutrient starvation and ER stress and found five genes (*Hnrnpd*, *Ogt, Srsf7*, *Ivns1abp* and *Hnrpdl*) to be spliced identically during either stress (Fig. 6A). These events were analyzed by compared percent spliced-in (PSI) values, as well as Sashimi plots generated via IGV, and were all validated by RT-PCR analysis RNA splice variant specific primer pairs (Fig. 6B and primer list in Table 1). Interestingly, all of these events involved the partial or full retention of intronic sequences that resulted in the introduction of an in-frame premature stop codon, producing a transcript to be degraded by nonsense mediated decay (NMD) according to the 55-bp rule [20]. Furthermore, these NMD-inducing events are evolutionarily conserved, as identified by other groups [9].

### Splicing of detained introns functions to regulate gene expression during cellular stress

A recent study identified a pool of mRNAs containing retained introns (referred to as detained introns or DIs in this study) that were spliced post-transcriptionally under stress conditions [21]. Interestingly, splicing of these DIs often resulted in a frameshifting event that generated potential NMD splice variants. Strikingly, several DI-containing genes reported in human cells by Boutz et al. were orthologs of genes found in our list (*Eny2*, *Ogt*, *Srsf7*, *Rbm39*, *Zfp326*, *Tra2a*, *Cdc16*, *Ivns1abp*, *Tex10*, *Hnrpdl*, *Slc35b1*, *Ufd1l* and *Clk4*), suggesting that ER stress might represent a physiological means to regulate splicing of DIs. Notably, all five of the AS events mentioned above occurring upon both ER stress and nutrient deprivation represented DIs identified by Boutz et al. to flank premature termination codon (PTC)-containing cassette sequences. Furthermore, extended manual analysis using IGV software revealed that this trend was consistent in RNA-binding proteins (*Rbm39*), U1 snRNP related (*Snrnp70*), U2 snRNP related (*Sf3b1*), core SR proteins (*Srsf3*, *Srsf6*, *Srsf5*), and other SR proteins (*Srrm1*, *Tra2a*), among others (Additional file 7: Figure S4). In agreement with the hypothesis that thapsigargin-regulated AS events corresponded to DIs, several AS events identified were predicted to generate potential NMD variants.

**Fig. 7:**
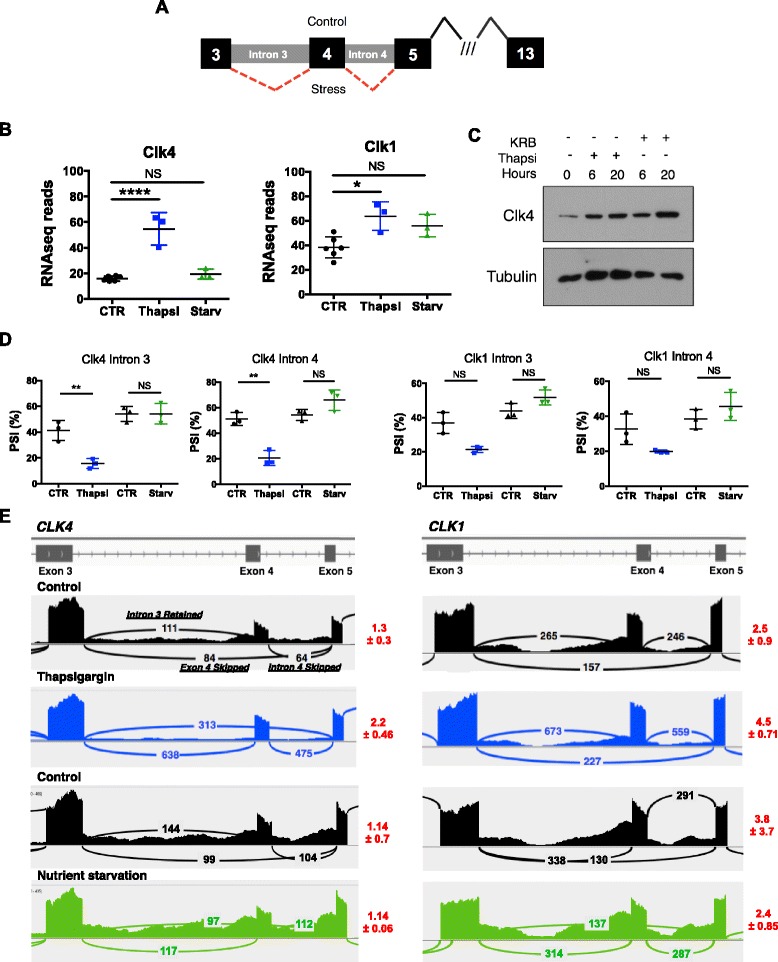
Splicing of retained introns in CLK4/1 upon ER stress. **a** Gene schematic showing splicing of detained introns (introns 3 and 4) upon control conditions vs. stress conditions (*red dotted line*). **b** Plots of expression values for *CLK1/4* upon thapsigargin treatment and nutrient starvation. **c** Western blot analysis of CLK4 protein upon thapsigargin and KRB stimulation for 6 or 20 h, as compared to tubulin loading control (**d**) PSI values for splicing of introns 3 and 4 of *CLK1/4*. **e** Sashimi plots representing the splicing of introns 3 and 4 of *CLK1/4*. Values in red represented the amount of DI splicing, calculated by taking the value of exon 4 skipped / (average of intron 3 retained, intron 4 retained), taken as an average of three biological replicates, with the standard deviation values. A higher value corresponds to more splicing of introns 3 and 4 and consequently, more retention of exon 4

Boutz et al. identified that the SR protein kinase CLK4 and its homologue CLK1 possess PTC-containing DIs (introns 3 and 4) that are spliced out to generate a functional translated protein (Fig. 7a). This provides a means of autoregulation and serves as the upstream regulator of splicing of the downstream DIs, as treatment with the CLK inhibitor CB19 affected the splicing of >300 DIs [21]. Consistent with the previous observations that the “fully spliced” (DI-lacking) *CLK1/4* transcripts encode a stable mRNA transcript, we observed an increase in expression of both *CLK4* and *CLK1* upon thapsigargin treatment (Fig. 7b). Furthermore, we observed an increase in CLK4 protein upon both thapsigargin and nutrient starvation (Fig. 7c). Interestingly, we observed significant splicing of introns 3 and 4 in both *CLK1* and *CLK4* upon thapsigargin treatment, analyzed by both PSI values and IGV Sashimi plots (Fig. 7d-e). Notably, there was no significant increase in *CLK1/4* mRNA expression, nor did we observe any change in PSI levels of intron 3 or 4 upon nutrient starvation, suggesting the presence of another upstream mechanism to regulate DI splicing upon starvation.

### Nutrient starvation and ER stress induce a subset of distinct AS events

**Fig. 8:**
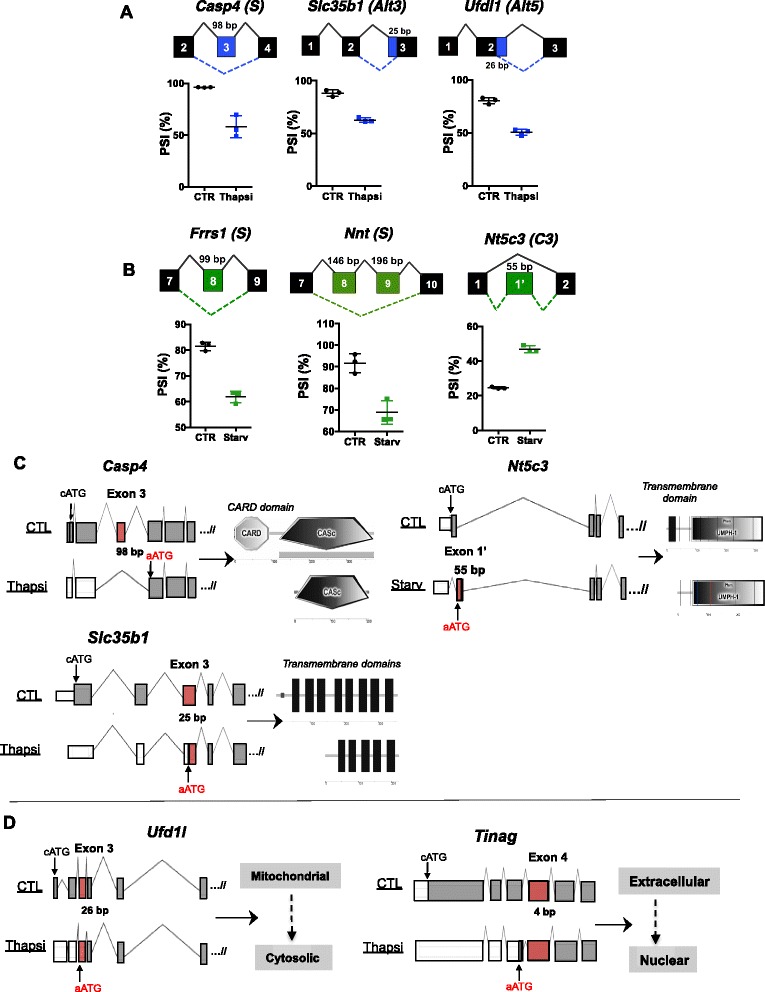
Validation of various alternative splicing events and in silico prediction of alternative ATG usage in splice variants induced upon cellular stress. **a-b** Six selected AS events induced upon thapsigargin treatment (*Casp4, Slc35b1, Ufdl1*) (**a**) and nutrient starvation (F*rrs, Nnt, Nt5c3*) (**b**). The type of AS event is indicated beside the gene name in parenthesis (S – skipping, Alt3 – alternative 3’, Alt5 – alternative 5’, C3 – complex type 3). Plots depicting the percent spliced-in (PSI) values for the AS events are shown. **c-d** Gene structures of full-length *Casp4, Slc35b1, Nt5c3, Tinag,* and *Ufd1l* using the canonical translation start sites (cATG) and their predicted splice variants induced by cellular stress (AS event highlighted in red). The alternative ATG (aATG) utilized by these variants were previously annotated by Aceview. The different protein domains encoded by the full-length and splice variants were analyzed using Simple Modular Architecture Research Tool (SMART) (**c**) and changes in protein localization were analyzed using PSORT (**d**)

In addition to a common signature of AS induced in enteroids during nutrient deprivation and ER stress, we noticed that most AS events (80/85 and 37/42 for ER stress and nutrient starvation, respectively) were stress-specific. Notably, the vast majority of these AS events that were unique to ER stress or nutrient starvation occurred in genes involved in diverse biosynthetic pathways. For example, AS events induced by stress occurred in various families of enzymes, such as cysteine-type peptidases (ie. *Usp15, Usp40*), mannosyltransferases (ie. *Alg9, Alg12*), isomerases (ie. *Rpe, Trub2*) and serine/threonine kinases (ie. *Bco2, Pla2g2a*). Interestingly, 3 of the identified AS targets in thapsigargin-stimulated cells are factors previously associated with ER stress response, including *Casp4*, *Slc35b1* and *Ufd1l* [22–24] (Fig. 8a, Additional file 8: Figure S5A). Furthermore, among the genes observed to undergo AS upon nutrient deprivation, several were involved enzymatic processes. Examples of these genes include the ferric-chelate reductase *Frrs1* and the NAD(P) transhydrogenase *Nnt,* which both undergo increased exon skipping upon starvation, and the 5’-nucleotidase *Nt5c3*, a gene for which we observed increased retention of a portion of intronic sequence (Fig. 8b, Additional file 8: Figure S5B). These results suggest that AS of genes involved in the response to stress contributes to a regulatory program associated with the cellular response to ER stress and nutrient deprivation.

### In silico prediction of the use of alternative translation start sites to generate novel isoforms

We noticed that many AS events induced by thapsigargin affected exons towards the 5’ end of the mRNAs, and database search (Aceview) identified the existence of potential alternative ATG (aATG) as potential translation initiation sites for 12 genes (*Casp4*, *Zfp326*, *Acot8*, *Ivns1abp*, *Tra2a*, *Rpl27a*, *G630016D24Rik*, *Slc35b1*, *Tex10*, *Rbm39*, *U2af1*, *BC024814*) among the 32 genes with the highest confidence AS (*p* ≥ 0.95, *DBS* = 0.15, *RN* = 10, data not shown), which could support the expression of shorter variants with truncated N-terminal ends (Additional file 9: Figure S6). All of these genes have been reported by Wilson et al. to utilize a novel aATG in both mouse and humans to maintain the proper frame of reading. Interestingly, many of the isoforms that would be generated upon usage of an alternative aATG have prominent changes in either protein domains (Fig. 8c) or protein localization (Fig. 8d). For example, upon skipping of the *Casp4* 98 bp exon 3, a novel variant using a previously annotated aATG just after the splicing event would generate an isoform lacking the entire caspase activation and recruitment domain (CARD domain). Splicing of the solute carrier *Slc35b1* in a manner that generates a 25 bp alternative 3’ event induced by thapsigargin and the 5’-nucleotidase *Nt5c3,* which undergoes an intron retention event of 55 bp upon nutrient starvation, resulted in the loss of 1 or more transmembrane domains and likely affects the subcellular localization of these proteins. Lastly, we predict that the thapsigargin-induced Alt3’ splicing of the ER stress gene *Ufd1l* and Alt5’ splicing of C1 family peptidase family gene *Tinag* would also utilize previously annotated aATGs to generate protein isoforms that are re-localized from the mitochondria to the cytosol, and the extracellular space to the nucleus, respectively [25].

## Discussion

Here we provide the first genome-wide snapshot of both the transcript level and AS reprogramming in response to cellular stress in primary IECs. The analysis presented here, which was conducted in an organoid model system derived from murine intestinal epithelial cells stimulated with two independent stresses – nutrient depletion and ER stress, provides a near-physiologically relevant investigation into the global response to metabolic stress in the gut.

None of the top GO terms associated with genes upregulated by nutrient starvation were directly related to a response to the starvation itself. Instead, our analysis revealed that a large number of metabolic genes were down-regulated by nutrient starvation, suggesting that short-term nutrient starvation does not turn on major stress response programs but, rather, slows down metabolic processes. This contrasts with the results obtained with ER stress, for which a clear upregulation of a transcriptional ER stress response program was identified. It should be noted that the results presented in this study are representative of the effect of thapsigargin and further investigation with additional ER stress inducing drugs such as tunicamycin would reaffirm these data in the global response to ER stress.

The transcriptional landscape regulated upon stress in primary intestinal cells reported here highlights a key link between cell stress and inflammatory immune responses. This likely stems from the fact that mucosal inflammation and chemokine-mediated recruitment of immune cells are both essential for the adaptation to stress within the intestinal epithelium. Indeed, the intestinal epithelium represents the first line of defense against pathogenic microbes and toxins infecting orally, and induction of an immune response during stress is essential for maintaining epithelial barrier integrity [26, 27]. Thus, our results highlight the importance of the ISR in the immune response of the intestinal epithelium, which is in agreement with reports linking stress signaling, such as XBP1-dependent pathways, with inflammatory bowel disease.

Because the intestinal epithelium contains a population of actively proliferating stem cells along with differentiated secretory/absorptive cells, we analyzed whether cellular stress affects proliferation and/or the stem-cell profile in epithelial organoids. Strikingly, we observed an overall decrease in proliferation measured by EdU incorporation and expression of proliferation genes (ie. Wnt signaling), as well as decreased ISC gene expression in thapsigargin treated samples. Interestingly, the opposite effect was observed during nutrient starvation and there was a significant increase in proliferation measured by EdU incorporation. While initially surprising, this finding likely highlights a transient response by the intestinal epithelium to increase proliferation in order to absorb more nutrients during short periods of nutrient deprivation via expansion of the intestinal absorptive surface. This explanation could explain the opposite effects of thapsigargin treatment and nutrient deprivation on proliferation. It is possible that this characteristic is specific to the intestinal epithelium and may not necessarily reflect cellular responses to nutrient starvation in other tissues. Additionally, consistent with previous reports that ER stress results in a loss of “stemness” in organoid cultures, we observed decreased expression of stem cell markers such as *Olfm4*, *Fzd2* and *Msi1* upon thapsigargin treatment, highlighting the fact that ER stress plays a key role in maintaining stem cell homeostasis in the intestinal epithelium [6]. Our analyses also identified a group of seven intestinal stem cell (ISC) genes that were regulated similarly by both stimulations, suggesting that these genes might represent the core group of genes involved in the cellular adaptation of ISCs to stress.

One prominent means of gene expression regulation among splicing factors is achieved by coupling AS with nonsense-mediated decay (NMD) [28, 29]. Specifically, highly conserved AS events promote inclusion of PTC-containing cassettes that consequently target transcripts for NMD; those AS events have been identified in SR proteins and various other RNA processing proteins [8, 9, 30]. We highlight in this study that these NMD-inducing AS events occur primarily in splicing-associated genes (ie. *Hnrnpd, Srsf7, Srsf3, Hnrpdl, U2af1, Tra2a*) and accumulate upon both nutrient starvation and ER stress. Consistent with our findings, a recent study reported that dozens of the mRNAs we report here contain detained introns flanking “NMD switch exons” that were spliced post-transcriptionally under stress conditions [21]. Furthermore, we report that expression of the NMD switch exon-containing transcript of the SR protein kinase CLK1/4, which was identified to be an upstream regulator of DI splicing, was also increased specifically upon ER stress [21, 31]. Notably, while ER stress and nutrient starvation induced identical AS events in various splicing genes, we believe that these events are regulated via a CLK1/4-independent mechanism upon nutrient starvation, as we did not observe splicing of CLK1/4 DIs. The stabilization of NMD target isoforms of various splicing and RNA processing genes can likely be attributed to translational blockade observed upon metabolic stress, which is known to inhibit NMD, as well as increased nuclear accumulation of unspliced transcripts upon stress [32, 33]. Nonetheless, these findings validate the existence of “NMD switch exons” AS events in intestinal organoids and are consistent with previous findings suggesting that the coupling of AS-NMD represents a key mechanism by which splicing genes are regulated.

In addition to the common signature of AS events leading to the generation of previously identified NMD targets transcripts, we observed a number of events specific to each stress that are instead predicted to use an alternative ATG in order to maintain the reading frame of the transcript, according to previous annotations [25]. These events were frequently located at the 5’ end of the transcript and often occurred in genes involved in specific responses to the presented stress, rather than splicing factors or RNA-processing proteins. The concept of frameshifting splice variants with alternative start codons is a largely overlooked form of AS that likely contributes significantly to transcriptome diversity. Future studies should aid in confirming the existence and functional significance of translated aATG isoforms to validate their role in the cellular response to stress.

## Conclusions

In summary, this study provides a genome-wide analysis on the gene expression patterns induced by metabolic stress in a murine intestinal organoid model. Cellular stresses like ER stress and nutrient starvation regulate the expression of genes involved in immune and defense, metabolism, proliferation and ISC activity. Additionally, we highlight a role for alternative splicing in shaping the stress response in the intestinal epithelium and reveal a potential mechanism for gene expression involving the coupling of AS to alternative translation start sites.

## Abbreviations

AA, amino acid; aATG, alternative start site; AS, alternative splicing; CHOP, cEBP homologous protein; DI, detained intron; ER, endoplasmic reticulum; GCN2, general controlled non-derepressible 2; GO, gene ontology; IGV, integrated genome viewer; ISC, intestinal stem cell; ISR, integrated stress response; KRB, Krebs-Ringer bicarbonate; NMD, nonsense mediated decay; PERK, PKR-like endoplasmic reticulum kinase; PKR, protein kinase R; PTC, premature stop codon; TA, transit-amplifying; UPR, unfolded protein response

## Additional files

Additional file 1: Table S1 cFPKM values for the global analysis of gene expression in murine enteroids upon either ER stress or nutrient starvation. (XLSX 5705 kb) Additional file 2: Table S2 Analysis of expression patterns of various genes upregulated or downregulated during metabolic stress, including those associated with metabolism and innate immunity. (XLS 60 kb) Additional file 3: Figure S1. Induction of a nutrient starvation-associated transcriptional program in intestinal organoids. (A) Venn diagram analysis of 240 genes upregulated more than 2.5 fold upon 4 h of nutrient starvation, categorized by gene function. (B) Gene list manually curated based on gene function from the group of 240 upregulated genes. (C) Venn diagram analysis of 165 genes downregulated more than 2.5 fold upon 4 h of nutrient starvation, categorized by gene function. (D) Total percent of genes with “unknown” function either upregulated or downregulated upon thapsigargin treatment or nutrient starvation. (E) Gene list manually curated based on gene function from the group of 165 downregulated genes. (PDF 381 kb) Additional file 4: Figure S2. Metabolic stress induces a downregulation of a common gene subset. (A) Venn diagram comparison of the genes commonly downregulated more than 2.5 fold upon nutrient starvation and thapsigargin treatment. Hypergeometric tests were used to calculate the P values for significance of overlaps. Scatterplot analysis of the genes transcriptionally downregulated (cutoff >0.4 fold) upon thapsigargin and nutrient starvation. Plots are displayed analyzing of the total number of genes (391) (B) or categorized by gene function (C). (D) Analysis of the gene function of the 18 genes commonly downregulated upon thapsigargin and nutrient starvation (cutoff >0.4 fold). (PDF 262 kb) Additional file 5: Figure S3. Analysis of the alternative splicing landscape in murine enteroids upon nutrient starvation. (A) Classification of AS events induced upon nutrient deprivation based on type of splicing event (skipping(S)/inclusion(I), complex 1 (C1), complex 3 (C3), alternative 3’ (Alt3), intron retention simple (IR-S), intron retention complex (IR-C), complex 2 (C2), alternative 5’ (Alt5)). Examples of both skipping and inclusion events are shown. (B) Scatterplot of the expression of the genes found to undergo AS upon nutrient starvation. Genes above the top red line represent a 2.5-fold increase in expression, while genes below the bottom red line represent genes down-regulated 2.5-fold. (C) Comparison of the proportion of frameshifting vs. non-frameshifting events within each category of AS type. (D) Examples of non-frameshifting AS events in Frrs1 (S) and 2410002O22Rik (S). Gene schematics showing the AS events in green, as well as the Sashimi plots obtained by IGV showing the total read numbers for each junction. (E) GO analysis of the gene group enrichment among the genes that underwent AS during nutrient starvation. (PDF 341 kb) Additional file 6: Table S3 Alternative splicing events in murine enteroids upon either ER stress or nutrient starvation. (XLSX 933 kb) Additional file 7: Figure S4. Metabolic stress promotes inclusion of PTC-containing poison cassettes in splicing factors/RNA processing genes. IGV plots displaying the alternative splicing events (highlighted) induced by nutrient starvation and ER stress. (PDF 180 kb) Additional file 8: Figure S5. Validation of AS events induced upon metabolic stress. Further validation of six selected AS events induced upon thapsigargin treatment (*Casp4, Slc35b1, Ufdl1*) (A) and nutrient starvation (F*rrs, Nnt, Nt5c3*) (B). Semi-quantitative RT-PCR analysis and sashimi plots generated via IGV are shown. (PDF 632 kb) Additional file 9: Figure S6. Overview of proposed mechanism of splicing events upon metabolic stress. Various splicing/RNA processing genes contain PTC-containing exons flanked by DIs, which can undergo splicing events resulting in the transcript being targeted to NMD. These NMD targets are stabilized during stress. Genes that are specific during stress may undergo AS coupled to the usage of an out of frame alternative translation start site (aATG) rather than the canonical start site (cATG), resulting in an N-terminal protein variant. (PDF 172 kb)
