# Supplementary material for: The transcriptional and splicing landscape of intestinal organoids undergoing nutrient starvation or endoplasmic reticulum stress
Source: BMC Genomics. 2016 Aug 26;17(1):680. doi: 10.1186/s12864-016-2999-1 (PMC5000506; doi:10.1186/s12864-016-2999-1)
Supplement: Additional file 3: Figure S1. — Induction of a nutrient starvation-associated transcriptional program in intestinal organoids. (A) Venn diagram analysis of 240 genes upregulated more than 2.5 fold upon 4 h of nutrient starvation, categorized by gene function. (B) Gene list manually curated based on gene function from the group of 240 upregulated genes. (C) Venn diagram analysis of 165 genes downregulated more than 2.5 fold upon 4 h of nutrient starvation, categorized by gene function. (D) Total percent of genes with “unknown” function either upregulated or downregulated upon thapsigargin treatment or nutrient starvation. (E) Gene list manually curated based on gene function from the group of 165 downregulated genes. (PDF 381 kb) [file 12864_2016_2999_MOESM3_ESM.pdf]

A

STARV >2.5X (N=240)

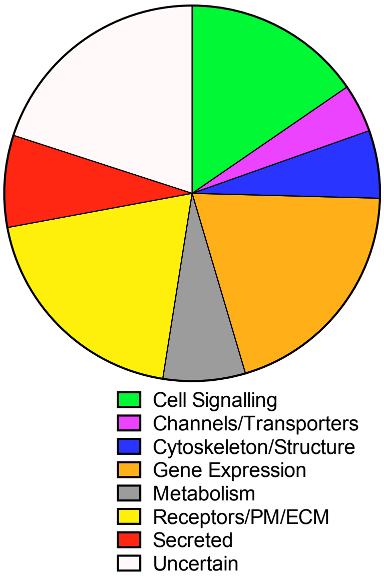

B

Transcription factors	Inflammation/Immune response
Atf3, Atf5, Egr1, Egr2, Egr3, Egr4, Fos, Fosb, Fosl1, Foxq1, Gbx1, Jun, Klf6, Maff, Nr4a3, Rax, Srf, Tceal7, Vgll3, Zmynd15	*Ccl20, Cd244, Cxcl1, Cxcl10, Edn1, Edn2, H2-M2, Il11, Lag3, Tff2, Tnfrsf11b, Tnfrsf25, Tnfsf15, Thbs1*
**Locomotion/Migration/Chemotaxis**	**Growth factor/Growth factor receptor**
Ccl20, Cyr61, Cxcl1, Cxcl10, Edn1, Edn2, Epha7, F3, Flrt3, Hbegf, Hgf, Itga1, Jam3, Plau, Plaur, Thbs1	*Ctgf, Cyr61, Edn1, Gdf9, Hbegf, Hgf, Slitrk6*

C

STARV <0.4X (N=165)

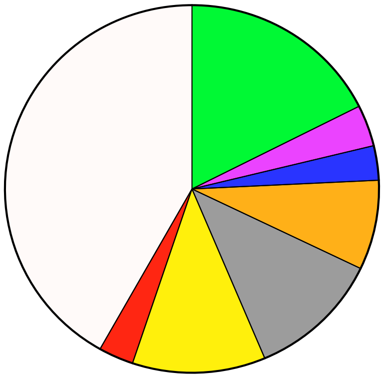

D

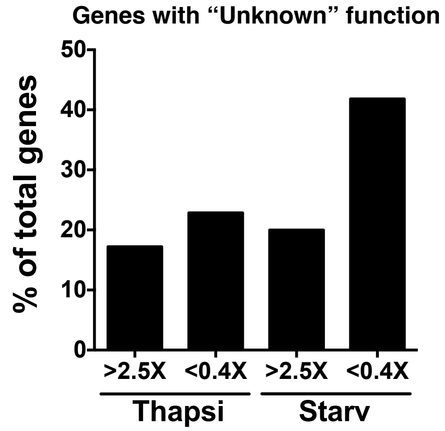

E

Cell cycle	Metabolism
Cdkn2a, Tigd3	*Alox12b, Arg2, Cbr3, Cyp1a1, G6pc, Gstm3, Haao, Haghl, Hpdl, Hrasls5, Insig1, Lipg, Lyrm7, Mb, Mns1, Nudt18, Sphk1, St6galnac4, Tpst1*
**Cellular Immunity**	
Card11, Ifitm6, Mx2, Nlrp10, Nlrp1b, Nos3, Noxa1	
