# Supplementary figures and images for: The transcriptional and splicing landscape of intestinal organoids undergoing nutrient starvation or endoplasmic reticulum stress
Source: BMC Genomics. 2016 Aug 26;17(1):680. doi: 10.1186/s12864-016-2999-1 (PMC5000506; doi:10.1186/s12864-016-2999-1)

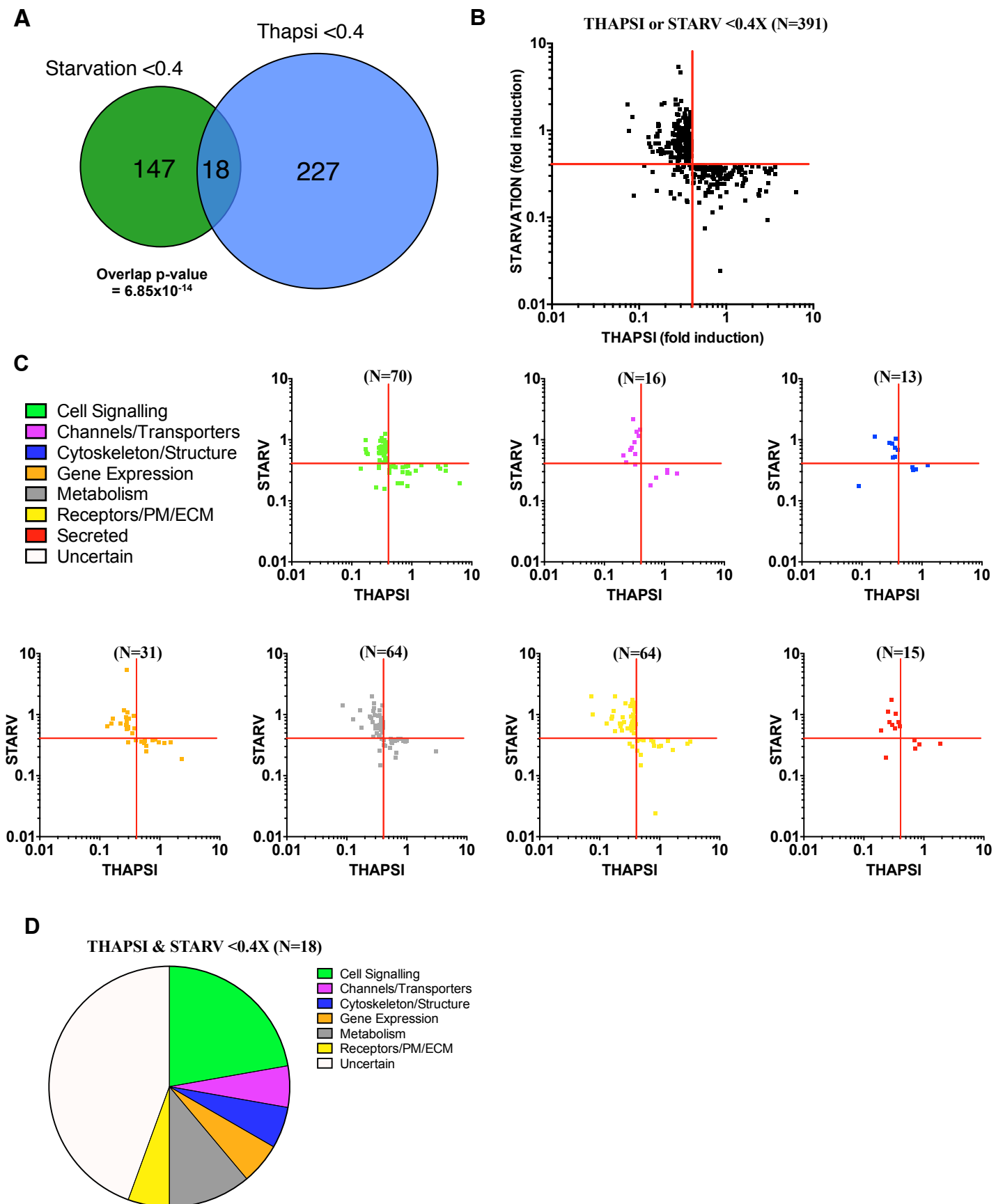

Supplement: Additional file 4: Figure S2. — Metabolic stress induces a downregulation of a common gene subset. (A) Venn diagram comparison of the genes commonly downregulated more than 2.5 fold upon nutrient starvation and thapsigargin treatment. Hypergeometric tests were used to calculate the P values for significance of overlaps. Scatterplot analysis of the genes transcriptionally downregulated (cutoff >0.4 fold) upon thapsigargin and nutrient starvation. Plots are displayed analyzing of the total number of genes (391) (B) or categorized by gene function (C). (D) Analysis of the gene function of the 18 genes commonly downregulated upon thapsigargin and nutrient starvation (cutoff >0.4 fold). (PDF 262 kb) [file 12864_2016_2999_MOESM4_ESM.pdf]

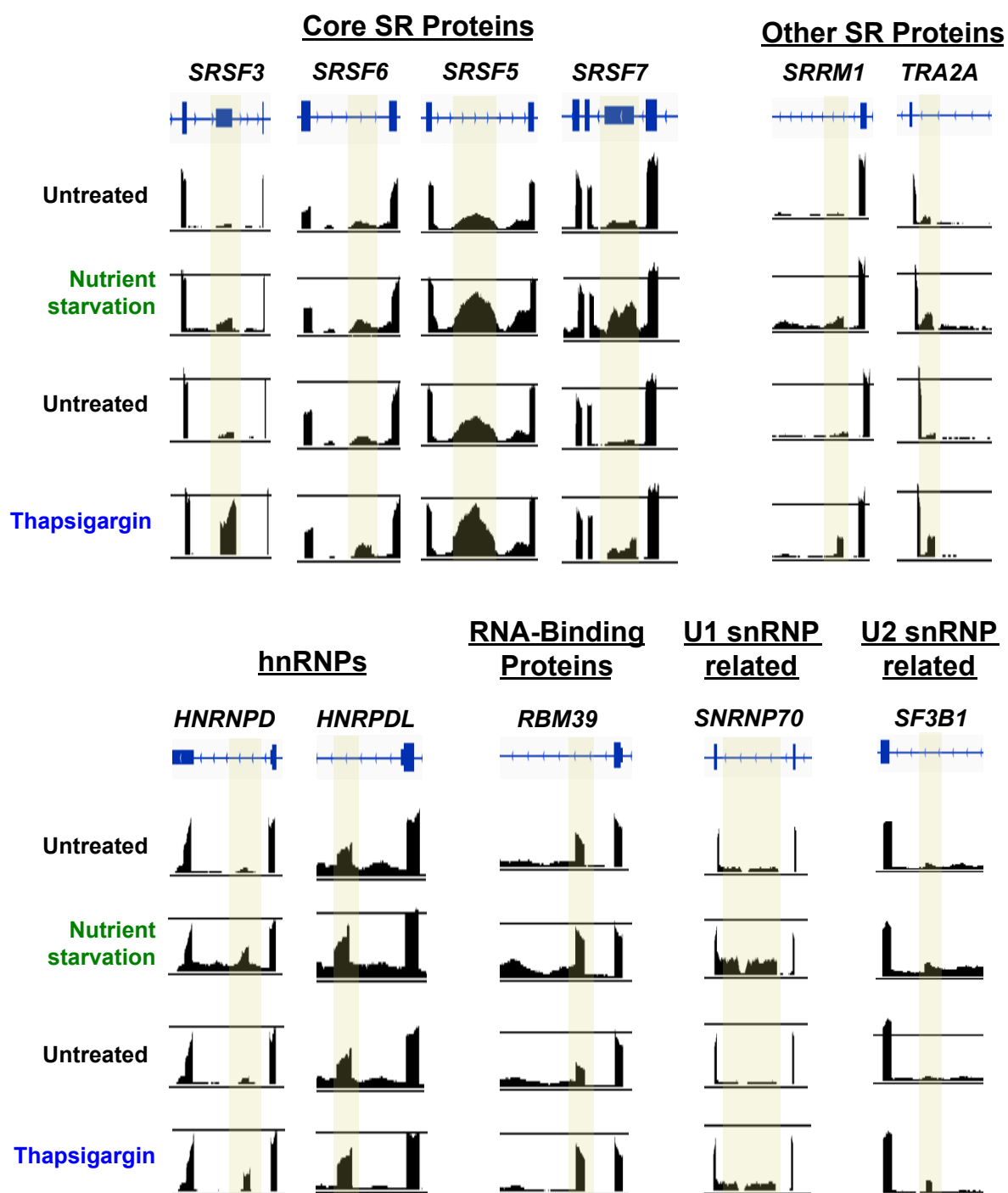

Supplement: Additional file 7: Figure S4. — Metabolic stress promotes inclusion of PTC-containing poison cassettes in splicing factors/RNA processing genes. IGV plots displaying the alternative splicing events (highlighted) induced by nutrient starvation and ER stress. (PDF 180 kb) [file 12864_2016_2999_MOESM7_ESM.pdf]

## Regulation of splicing genes

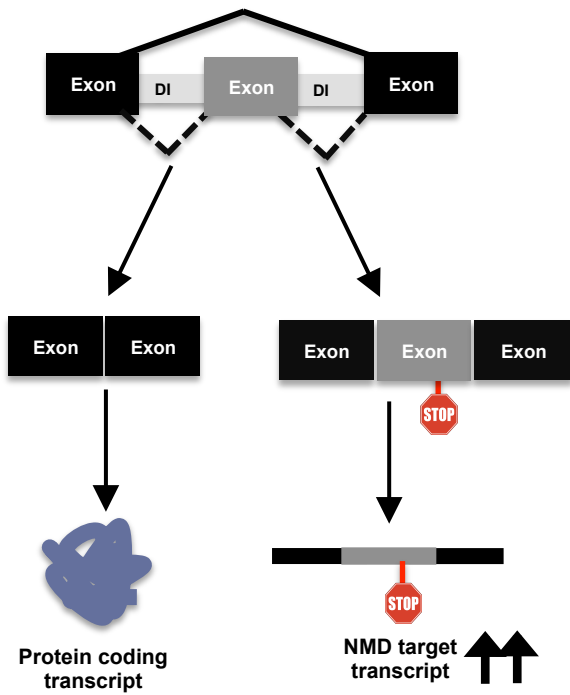

## Regulation of stress-specific genes

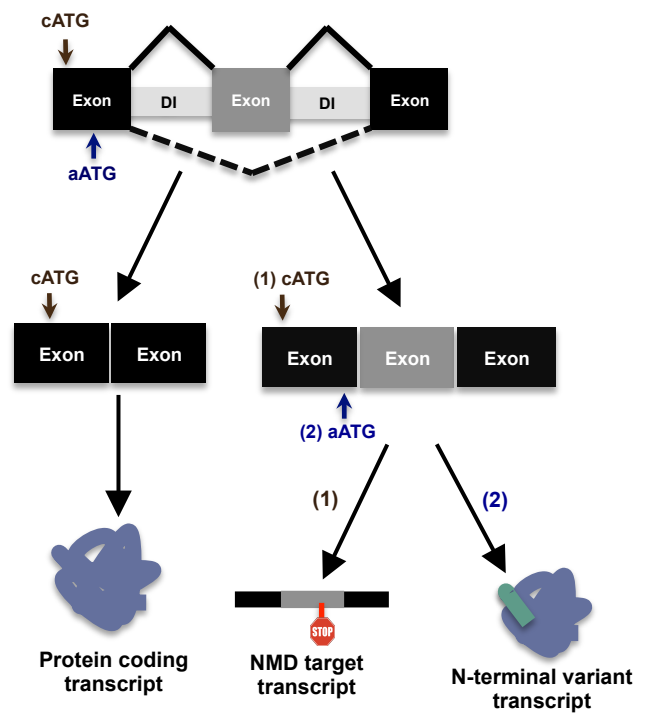

Supplement: Additional file 8: Figure S5. — Validation of AS events induced upon metabolic stress. Further validation of six selected AS events induced upon thapsigargin treatment (Casp4, Slc35b1, Ufdl1) (A) and nutrient starvation (Frrs, Nnt, Nt5c3) (B). Semi-quantitative RT-PCR analysis and sashimi plots generated via IGV are shown. (PDF 632 kb) [file 12864_2016_2999_MOESM8_ESM.pdf]

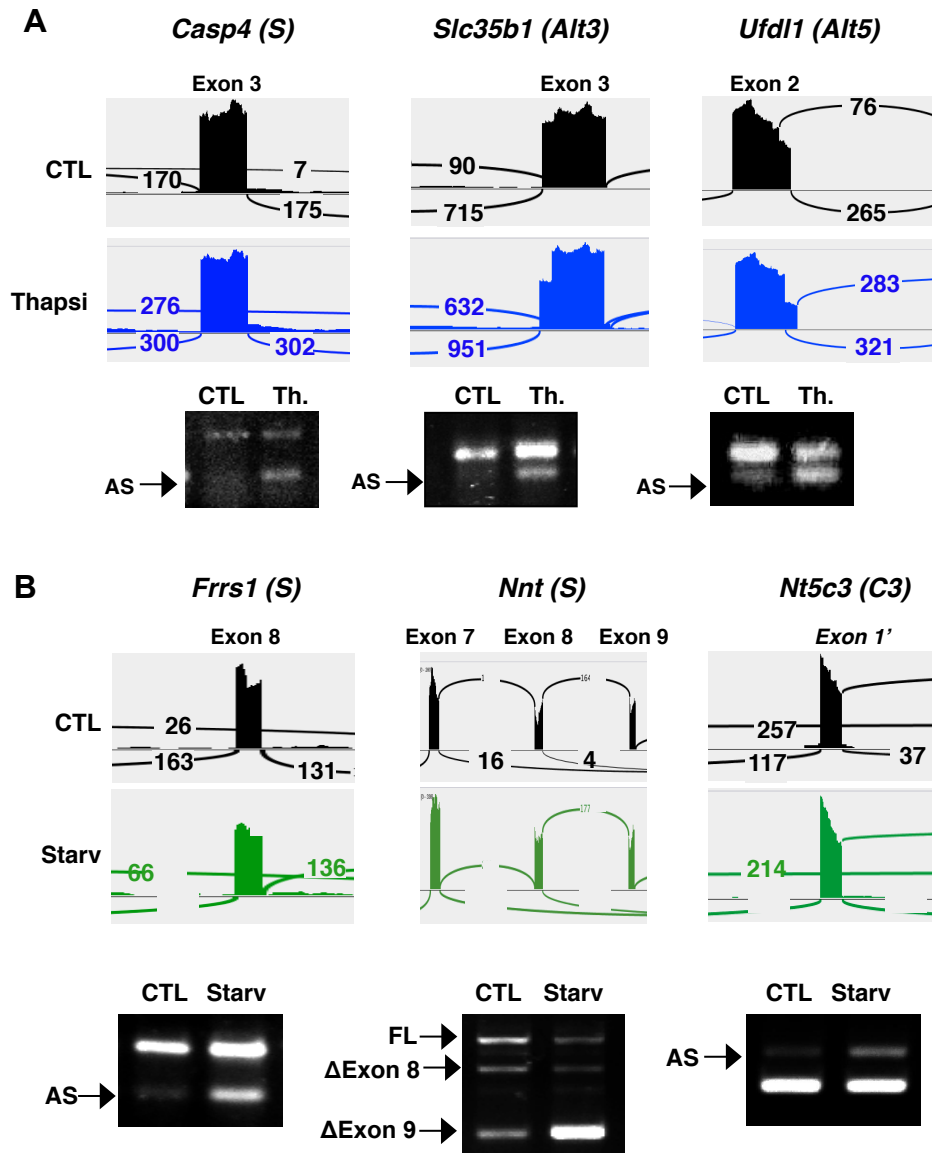

Supplement: Additional file 9: Figure S6. — Overview of proposed mechanism of splicing events upon metabolic stress. Various splicing/RNA processing genes contain PTC-containing exons flanked by DIs, which can undergo splicing events resulting in the transcript being targeted to NMD. These NMD targets are stabilized during stress. Genes that are specific during stress may undergo AS coupled to the usage of an out of frame alternative translation start site (aATG) rather than the canonical start site (cATG), resulting in an N-terminal protein variant. (PDF 172 kb) [file 12864_2016_2999_MOESM9_ESM.pdf]
