# Supplementary material for: The transcriptional and splicing landscape of intestinal organoids undergoing nutrient starvation or endoplasmic reticulum stress
Source: BMC Genomics. 2016 Aug 26;17(1):680. doi: 10.1186/s12864-016-2999-1 (PMC5000506; doi:10.1186/s12864-016-2999-1)
Supplement: Additional file 5: Figure S3. — Analysis of the alternative splicing landscape in murine enteroids upon nutrient starvation. (A) Classification of AS events induced upon nutrient deprivation based on type of splicing event (skipping(S)/inclusion(I), complex 1 (C1), complex 3 (C3), alternative 3’ (Alt3), intron retention simple (IR-S), intron retention complex (IR-C), complex 2 (C2), alternative 5’ (Alt5)). Examples of both skipping and inclusion events are shown. (B) Scatterplot of the expression of the genes found to undergo AS upon nutrient starvation. Genes above the top red line represent a 2.5-fold increase in expression, while genes below the bottom red line represent genes down-regulated 2.5-fold. (C) Comparison of the proportion of frameshifting vs. non-frameshifting events within each category of AS type. (D) Examples of non-frameshifting AS events in Frrs1 (S) and 2410002O22Rik (S). Gene schematics showing the AS events in green, as well as the Sashimi plots obtained by IGV showing the total read numbers for each junction. (E) GO analysis of the gene group enrichment among the genes that underwent AS during nutrient starvation. (PDF 341 kb) [file 12864_2016_2999_MOESM5_ESM.pdf]

Figure S3

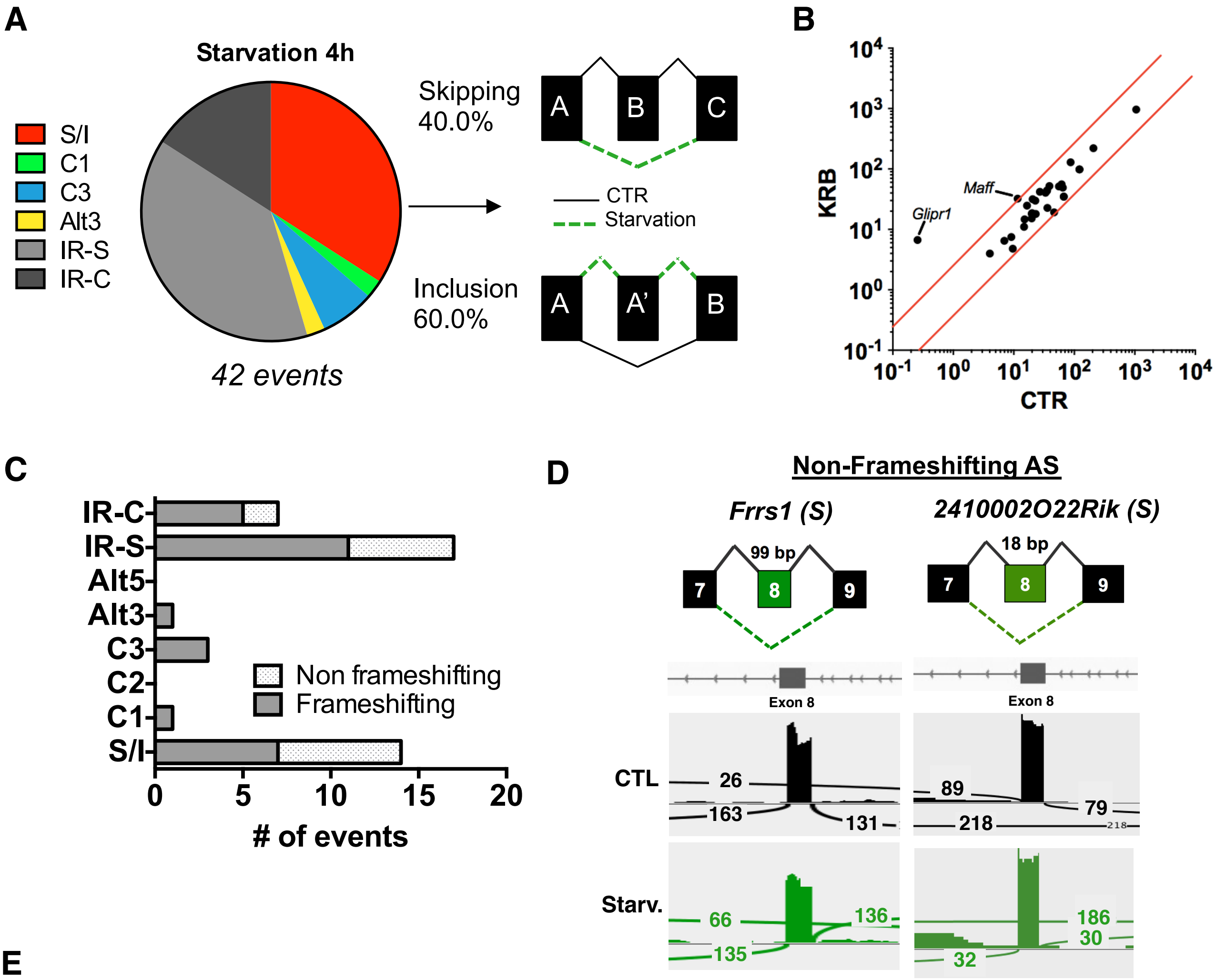

E

GO Biological process	GO #	Gene	p value
Positive regulation of histone methylation	0031062	*Ogt, Rnf20*	$6.4\ 10^{-4}$
RNA splicing	0008380	*Srsf7, Snrnp70, Hnrnpd, Ivns1abp, Smndc1,* (*Hnrpdl*)	$8.7\ 10^{-4}$
Cellular response to extracellular stimulus	0031668	*Gsdmd, Gas2l1*	$1.2\ 10^{-2}$
